# Miliary Tuberculosis Diagnosed After Repeated Sputum Smear Examinations Despite Negative Results at a Referral Tuberculosis Center: The Importance of Maintaining Clinical Suspicion

**DOI:** 10.1002/jgf2.70164

**Published:** 2026-08-24

**Authors:** Kento Kabeya, Kousuke Mouri, Hidehiro Someko

**Affiliations:** ^1^ Department of Internal Medicine Nagoya Tokushukai General Hospital Kasugai Japan; ^2^ SRWS‐PSG Osaka Japan

**Keywords:** anchoring bias, diagnostic delay, diagnostic momentum, disseminated tuberculosis, tuberculous spondylitis

## Abstract

An 88‐year‐old woman with back pain was found to have destructive changes at T9/T10 and diffuse micronodular pulmonary opacities suggestive of miliary tuberculosis. At two referring hospitals, six sputum smears and two interferon‐gamma release assays were negative. Because the clinical and radiological findings remained highly suggestive of tuberculosis, three additional sputum smears were obtained after admission, leading to rapid microbiological confirmation that avoided the need for invasive diagnostic procedures. This case highlights the importance of maintaining clinical suspicion and repeating sputum examination despite extensive negative evaluation elsewhere.

## 1 Introduction

Miliary tuberculosis (TB) is a disseminated form of *Mycobacterium tuberculosis* infection characterized radiologically by diffuse micronodular pulmonary lesions [1]. Because cavitary lesions are uncommon, the airway bacillary burden is often low, and reported cumulative sputum smear positivity rates across serial examinations in miliary tuberculosis are approximately 40%–50%—substantially lower than in typical pulmonary TB [2, 3]. Interferon‐gamma release assays (IGRAs) are similarly prone to false‐negative results in elderly and immunocompromised patients [4].

When initial smears are repeatedly negative, clinicians face a consequential choice: proceed to invasive sampling—bronchoalveolar lavage, gastric lavage, or extrapulmonary biopsy—or apply reflective reasoning and repeat sputum examination. This decision is further complicated when the patient arrives having already undergone extensive evaluation elsewhere. Because these repeatedly negative results accumulate as a patient is referred between institutions, they can foster anchoring bias—an over‐reliance on earlier findings that is insufficiently revised as new information emerges—and diagnostic momentum, in which a prior interpretation is carried forward across clinicians and treated as established [5]. A recognized countermeasure is reflective reasoning: deliberately disengaging from the inherited impression to re‐evaluate the current syndrome, pre‐test probability, and the basic diagnostic sequence. Yet the fundamental principle remains: sputum smear is the appropriate first step when pulmonary TB is suspected [6]. Evidence specific to miliary tuberculosis is limited; however, a systematic review of 37 studies of pulmonary tuberculosis found that, among patients ultimately identified as smear‐positive, 85.8% were detected with the first specimen, with incremental yields of 11.9% and 2.3% from the second and third specimens, respectively [7].

We report an 88‐year‐old woman with miliary TB and tuberculous spondylitis first microbiologically confirmed at our hospital on the third sputum smear (the ninth overall) within 24 h of admission, after six negative smears and two negative IGRAs at two referring hospitals. This case illustrates that strong clinical suspicion must override prior negative results, and that the diagnostic process warrants restarting, using reflective reasoning, when miliary‐pattern imaging remains unexplained.

## 2 Case Presentation

**FIGURE 1:**
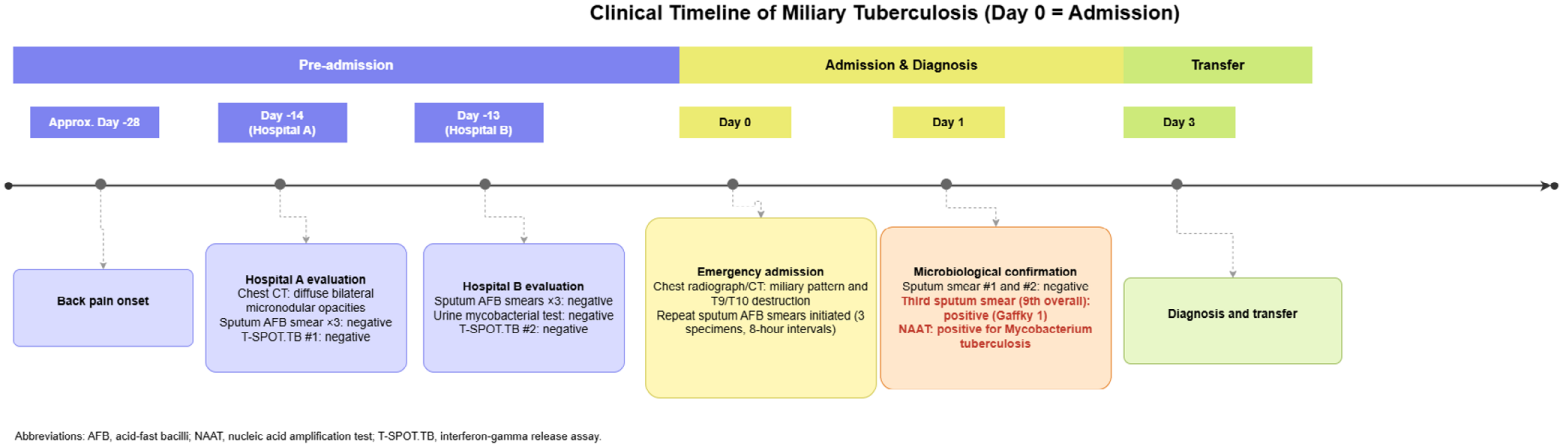
Clinical timeline of symptom onset, diagnostic workup, and hospital transfer (Day 0 = Admission). The patient had back pain for approximately 4 weeks before admission. At Hospital A and Hospital B, six sputum AFB smears and two T‐SPOT.TB assays were negative before admission.

An 88‐year‐old woman was brought to our emergency department by ambulance after she became unable to walk at home. She had first noticed back pain 2 weeks earlier, prompting a visit to a local clinic. According to the referral report, spinal magnetic resonance imaging suggested destructive changes at the T9/T10 vertebral level. She was referred to Hospital A for further evaluation, where chest computed tomography demonstrated diffuse bilateral micronodular opacities. Miliary tuberculosis was suspected, and three sputum acid‐fast bacilli (AFB) smears and an interferon‐gamma release assay (T‐SPOT.TB) were performed, all of which were negative. Hospital A had not submitted specimens for mycobacterial culture. Thirteen days before admission, she underwent further evaluation at Hospital B, where three additional sputum AFB smears, urine acid‐fast bacilli smear examination, and repeat T‐SPOT.TB were again negative; the sputum culture collected at Hospital B subsequently grew *Mycobacterium tuberculosis* , but this result was not yet available at the time of her admission to our hospital. The clinical course is summarized in Figure 1.

Her medical history included hypertension, overactive bladder, osteoporosis, previous cataract surgery, and lumbar compression fracture. She had no family history of tuberculosis, no history of overseas travel, and had not resided in a care home or residential care facility. She had no known drug allergies, no smoking history, and no alcohol use. She had no history of oral fluoroquinolone use, and she had been independent in activities of daily living before this illness. Her regular medications were sacubitril/valsartan, rabeprazole, vibegron, bisoprolol fumarate, nifedipine, and aspirin.

On arrival, her vital signs were: blood pressure 178/100 mmHg, pulse 75 beats/min, respiratory rate 20 breaths/min, temperature 37.1°C, and oxygen saturation 95% on room air. She was alert (Glasgow Coma Scale E4V5M6). Cardiac examination revealed a grade II/VI systolic murmur at the mitral area, and fine crackles were heard over both lower lung fields. No motor weakness was observed in the extremities, and no neurological deficits were identified.

Laboratory investigations revealed a white blood cell count of 6.1 × 10^3^/μL with neutrophil predominance, hemoglobin 10.1 g/dL, albumin 3.1 g/dL, lactate dehydrogenase 295 U/L, and C‐reactive protein 8.40 mg/dL. Renal function was preserved.

**FIGURE 2:**
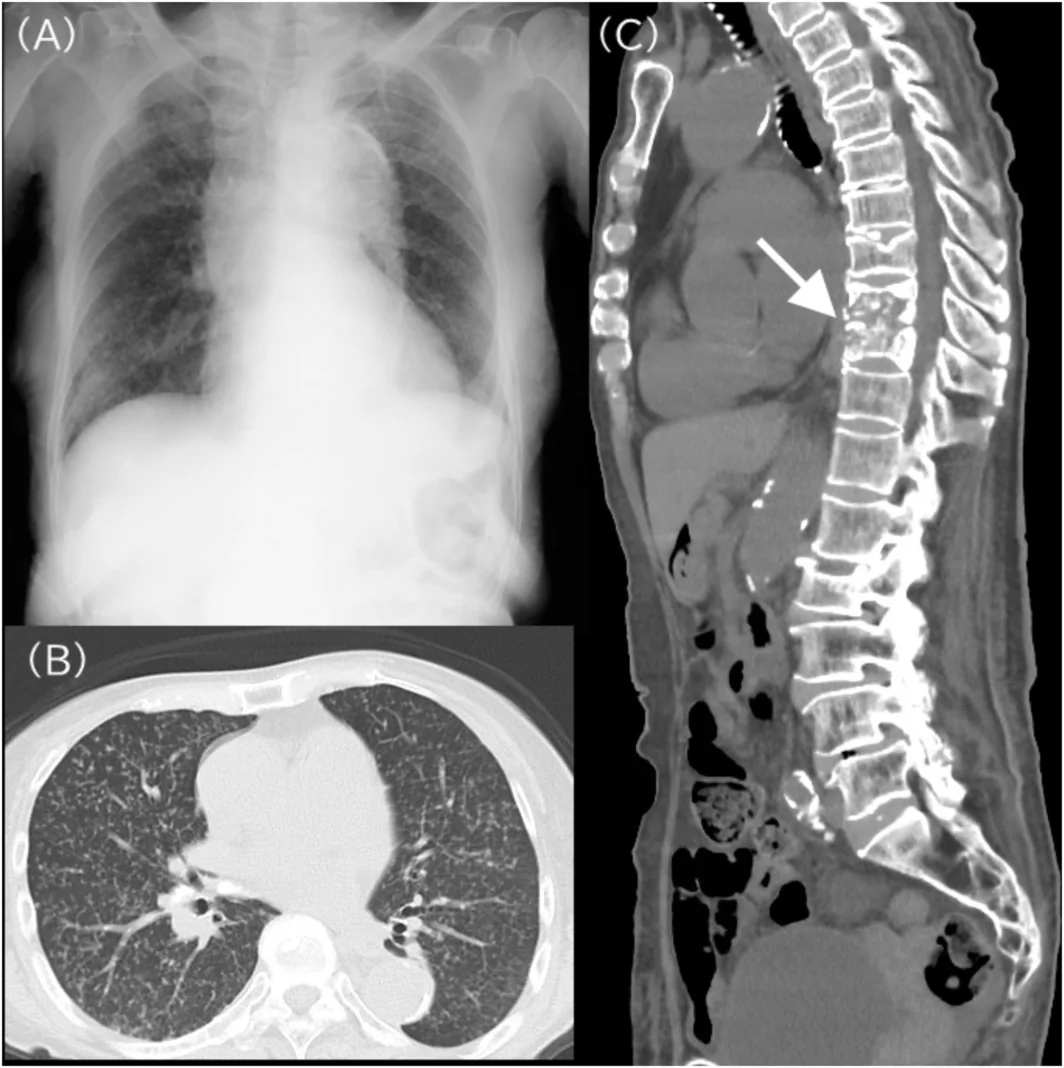
Chest radiography and computed tomography findings on admission. (A) Chest radiograph showing diffuse bilateral fine nodular opacities. (B) Chest computed tomography demonstrating diffuse bilateral finely granular pulmonary lesions consistent with a miliary pattern. (C) Sagittal computed tomography image showing destructive changes at the T9/T10 vertebral level (arrow).

Chest radiography showed diffuse bilateral fine nodular opacities. Computed tomography showed prominent osteolytic destruction, predominantly involving the T9 vertebral body, with destruction of the inferior endplate of T9 and the superior endplate of T10. Mild narrowing of the T9/T10 intervertebral disc space and increased adjacent paravertebral soft‐tissue density, representing reactive inflammatory tissue without fluid‐density abscess formation, were also observed. No obvious epidural extension or spinal canal compromise was evident on CT (Figure 2A–C). Because spinal magnetic resonance imaging performed at the prior clinic could not be obtained for direct review, detailed assessment of marrow edema, epidural inflammation, and disc signal abnormalities was limited. Despite six prior negative smears and two negative IGRAs, the imaging findings and subacute clinical course led us to strongly suspect miliary tuberculosis with concomitant tuberculous spondylitis. Because the syndromic findings sustained a high pre‐test probability of miliary tuberculosis despite the prior negative results, we did not treat the prior negative results as definitive and elected to repeat non‐invasive testing before considering invasive sampling. Sputum AFB smear examinations were repeated at 8‐h intervals for three consecutive specimens, with blood mycobacterial culture and repeat T‐SPOT.TB performed concurrently. At our hospital, all three respiratory specimens were obtained by suction; sputum induction, chest physiotherapy, and other special collection techniques were not used. Detailed information regarding sputum collection methods at the referring hospitals was unavailable.

The first, second, and third specimens were collected in the evening, during the night, and in the early morning, respectively, at 8‐h intervals. The first two specimens were smear‐negative, whereas the third specimen (the ninth overall) was smear‐positive (Gaffky scale 1). Nucleic acid amplification testing was positive for *Mycobacterium tuberculosis* in all three specimens, including the first two smear‐negative specimens; cultures from all three specimens subsequently became positive. Repeat T‐SPOT.TB and blood mycobacterial culture remained negative. A diagnosis of miliary tuberculosis was established, with a clinical diagnosis of tuberculous spondylitis; no neurological deficits, paraspinal abscess, or epidural abscess were identified. The patient was transferred to Hospital B on day 3 for ongoing management at a designated tuberculosis referral center.

## 3 Discussion

We report an elderly woman in whom miliary tuberculosis was first microbiologically confirmed at our hospital via smear examination completed within 24 h (the third specimen obtained at our hospital, and the ninth overall), which was followed by subsequent confirmation of *Mycobacterium tuberculosis* by nucleic acid amplification testing (NAAT) and culture. Although the sputum culture collected at Hospital B 13 days prior to admission ultimately grew *Mycobacterium tuberculosis* , this result was unavailable at the time of her presentation. Repeating the sputum examination at our hospital provided microbiological confirmation within 24 h of admission, allowing prompt infection‐control decisions and timely transfer to a designated tuberculosis center. Thus, the educational message of this case is that reassessment and repeat non‐invasive sampling can accelerate diagnosis when pending results are unavailable and the clinical suspicion remains high. Empiric treatment was not initiated at our hospital because the patient remained clinically stable without respiratory failure, hemodynamic instability, or central nervous system involvement, and prompt transfer to a designated tuberculosis center was possible.

Previously negative results at referring institutions should not deter repeat sputum smear examination when miliary tuberculosis remains strongly suspected on clinical and radiological grounds. The diagnostic reasoning in this case was based on the overall clinical syndrome rather than on any single test result. Subacute back pain with destructive changes at T9/T10 suggested chronic infectious spondylitis, whereas the simultaneous diffuse micronodular pulmonary pattern suggested hematogenous dissemination. This combination was difficult to explain with a localized degenerative or malignant process alone, and the diffuse bilateral micronodular pattern suggestive of random hematogenous dissemination most strongly increased the pre‐test probability of miliary tuberculosis, despite repeated negative sputum smears and IGRAs. On that basis, we deliberately re‐evaluated the diagnostic process rather than accepting the negative results obtained at prior institutions as definitive. Prior negative results may foster anchoring bias and diagnostic momentum [5], particularly when patients move across institutions, prompting premature de‐escalation of isolation measures or diagnostic workup. Premature closure—the tendency to accept a diagnosis or to stop the diagnostic process before it has been fully verified [8]—may also contribute to delayed tuberculosis diagnosis. Whether it operated here cannot be determined from the available records. Because the contribution of any single bias at the referring institutions cannot be established retrospectively, the more actionable lesson concerns the receiving clinician's task: to avoid carrying forward accumulated negative results as established, and to re‐evaluate the syndrome on its own terms.

Typical radiological features of tuberculous spondylitis include osteolytic bone destruction, endplate erosion, and relative preservation of the disc space until late stages [9]. In our patient, the prominent osteolytic destruction of the T9 vertebral body with endplate erosion strongly suggested tuberculous spondylitis and prompted further diagnostic efforts. The differential diagnosis of the destructive T9/T10 lesion included pyogenic spondylitis, primary or metastatic spinal malignancy, multiple myeloma, and changes related to vertebral compression fractures. Spinal biopsy was deferred because the miliary pulmonary pattern and vertebral lesion formed a coherent disseminated tuberculosis syndrome, and *M. tuberculosis* was microbiologically confirmed from respiratory specimens. No neurological deficits, paraspinal abscess (despite reactive soft‐tissue density on CT), or epidural abscess were present. Spinal biopsy would have been reconsidered if the subsequent clinical or radiologic course, or the response to anti‐tuberculosis therapy, had been inconsistent with this diagnosis.

The present case also illustrates the practical value of 8‐h interval sputum collection. CDC infection‐control guidance for suspected pulmonary or laryngeal tuberculosis states that three sputum specimens for AFB smear should be collected 8–24 h apart, with at least one early‐morning specimen [10]. Although the incremental yield from a third specimen is statistically small in routine cohorts, cumulative yield increases with optimized serial sampling and high‐quality collection. In our patient, this rapid protocol allowed completion of repeat sputum examination within 24 h of admission, and the third specimen was smear‐positive, enabling prompt microbiological confirmation. Detailed information regarding the collection technique, volume, quality, and exact timing of the six sputum specimens obtained at Hospitals A and B was unavailable. Because these examinations occurred within a relatively short period before admission, the subsequent positive smear cannot be confidently attributed to increasing bacillary burden over time. Differences in specimen quality, collection technique, timing, and random sampling variation remain possible explanations.

False‐negative IGRA results in older patients may reflect immunosenescence, including reduced number and function of antigen‐responsive T cells and diminished interferon‐gamma production. Disseminated or severe tuberculosis may itself suppress cell‐mediated immunity and produce functional T‐cell anergy. Lymphopenia and comorbid conditions associated with impaired cellular immunity, such as HIV infection, malignancy, and connective tissue disease, have also been associated with false‐negative results; low hemoglobin has been reported as an additional associated factor. Thus, repeated negative IGRAs do not exclude active disseminated tuberculosis in an elderly patient when the clinical and radiologic probability remains high.

Repeat testing should be guided by pre‐test probability rather than performed reflexively. When the probability of tuberculosis is low, excessive repetition may delay alternative diagnoses, consume healthcare resources, prolong isolation, increase patient burden, and create confusion through false‐positive results or incidental detection of non‐tuberculous mycobacteria. Repeat non‐invasive sampling is most defensible when the clinical and radiologic probability remains high, the quality, timing, or method of prior testing is uncertain, and optimized specimens can be obtained promptly.

In managing suspected miliary tuberculosis with comorbid spondylitis when initial microbiologic testing is negative, clinicians must carefully balance the diagnostic yield of invasive procedures against procedural risks. In our patient, invasive diagnostics (such as bronchoalveolar lavage [BAL] or vertebral biopsy) were initially deferred because she was clinically stable, and repeat non‐invasive sputum examinations could be performed rapidly within 24 h. Had all three sputum specimens at our institution returned negative, the next diagnostic steps would have been determined by her clinical status and institutional capabilities. For a patient who remained hemodynamically stable without progressive respiratory decline, vertebral biopsy would have been the preferred secondary option due to the presence of destructive spondylitis, as it provides tissue confirmation with relatively low procedural risk. Conversely, had her respiratory status deteriorated, BAL would have been pursued. Ideally, controlled BAL during intubation would be the safer choice if mechanical ventilation became necessary. Given the lack of specialized manpower to safely perform urgent bronchoscopy under strict airborne‐infection precautions at our community hospital, these invasive procedures would have been deferred until after her prompt transfer to a designated tuberculosis referral center. Establishing such pre‐specified escalation criteria—defining trigger conditions, preferred procedures, and safety thresholds—is critical for guiding clinical decisions when non‐invasive tests remain non‐diagnostic.

## 4 Conclusion

Negative microbiologic tests do not exclude miliary tuberculosis when the clinical syndrome and radiologic findings remain strongly suggestive. When the syndrome sustains a high pre‐test probability, clinicians should not treat previous negative results as definitive; reassessing the pre‐test probability and promptly repeating optimized non‐invasive sampling can confirm the diagnosis rapidly and non‐invasively, while thresholds for invasive sampling are defined in advance.

## Author Contributions

**Kousuke Mouri:** writing – review and editing, project administration. **Hidehiro Someko:** writing – review and editing, supervision. **Kento Kabeya:** conceptualization, writing – original draft.

## Consent

The authors have obtained patient consent.

## Conflicts of Interest

The authors declare no conflicts of interest.

## Data Availability

Research data are not shared.
